# Imported cases and minimum temperature drive dengue transmission in Guangzhou, China: evidence from ARIMAX model

**DOI:** 10.1017/S0950268818001176

**Published:** 2018-05-21

**Authors:** Q. L. Jing, Q. Cheng, J. M. Marshall, W. B. Hu, Z. C. Yang, J. H. Lu

**Affiliations:** 1 Department of Medical Statistics and Epidemiology, School of Public Health, Sun Yat-sen University, Guangzhou, People's Republic of China; 2 Ministry of Education Key Laboratory for Earth System Modeling, Center for Earth System Science, Tsinghua University, Beijing, People's Republic of China; 3 Biostatistics and Epidemiology, School of Public Health, University of California, Berkeley, California, USA; 4 School of Public Health and Social Work, Queensland University of Technology, Brisbane, Australia; 5 Department of Infectious Diseases, Guangzhou Center for Disease Control and Prevention, Guangzhou, People's Republic of China

**Keywords:** Dengue fever, local transmission, epidemiology, ARIMAX

## Abstract

Dengue is the fastest spreading mosquito-transmitted disease in the world. In China, Guangzhou City is believed to be the most important epicenter of dengue outbreaks although the transmission patterns are still poorly understood. We developed an autoregressive integrated moving average model incorporating external regressors to examine the association between the monthly number of locally acquired dengue infections and imported cases, mosquito densities, temperature and precipitation in Guangzhou. In multivariate analysis, imported cases and minimum temperature (both at lag 0) were both associated with the number of locally acquired infections (*P* < 0.05). This multivariate model performed best, featuring the lowest fitting root mean squared error (RMSE) (0.7520), AIC (393.7854) and test RMSE (0.6445), as well as the best effect in model validation for testing outbreak with a sensitivity of 1.0000, a specificity of 0.7368 and a consistency rate of 0.7917. Our findings suggest that imported cases and minimum temperature are two key determinants of dengue local transmission in Guangzhou. The modelling method can be used to predict dengue transmission in non-endemic countries and to inform dengue prevention and control strategies.

## Introduction

Dengue is the fastest spreading mosquito-borne viral disease worldwide and is primarily transmitted by *Aedes albopictus* and *Aedes aegypti*. It affects 3.6 billion people, with 96 million out of approximately 390 million infections annually resulting in symptomatic infection [1]. Dengue circulates in more than 100 countries each year and has the great impact in the Americas, Asia, Africa and Australia [2], imposing a great economic burden on affected areas with accounting for approximately 1 billion US dollars in annual economic cost for Southeast Asian countries [3].

The causative agent of dengue is dengue virus (DENV), which is antigenically divided into four serotypes (DENV-1, DENV-2, DENV-3 and DENV-4) [4]. The spectrum of dengue infection ranges from inapparent infection, non-specific febrile illness and self-limited dengue fever (DF) to severe dengue, which manifested by dengue hemorrhagic fever (DHF) and dengue shock syndrome (DSS). A second infection caused by a different serotype can increase the risk for severe disease mainly due to antibody-dependent enhancement (ADE) [5]. Rapid fluid loss into tissue spaces can lead to death from hemoconcentration and hypotension [2]. At present, the licensed vaccine must be combined with effective vector control to prevent large-scale outbreaks due to its modest efficacy [6].

Dengue was first identified in mainland China in 1978 from Foshan City after 1949, that is adjacent to Guangzhou [7] and has been a notifiable disease since 1 September 1989 [8]. Dengue transmission mostly occurred in Guangzhou City with more than 50% of the cases in mainland China reported since 1989 [9]. All four serotypes had circulated in Guangzhou since 1978 with one serotype dominating each transmission year [9]. Reported indigenous cases meaning locally acquired dengue infections in Guangzhou soared dramatically beyond expectations in 2014, with 37 340 indigenous cases that accounted for approximately 80% of the total reported cases in mainland China [10]. However, the critical risk factors of dengue transmission in Guangzhou are still poorly understood.

Statistical models indicate that dengue incidence on a local scale is associated with imported cases also named travel-related dengue infections, vector densities, climate variables, socioeconomic and environmental factors, population immune status and human movement [11–15]. However, the exact relationship between the risk factors and dengue incidence varies by location; for instance, a variable can increase the dengue risk in some districts but decrease it in other areas [16]. Early importation and larger numbers of imported cases in the calendar year have been identified as the most important determinants underlying dengue transmission in China [10], Japan [17] and Australia [18]. Most studies suggest that precipitation is positively correlated with dengue risk in tropical countries; however, a few epidemics have also been documented in areas with low rainfall or larval indices, such as Thailand in 1987 [19] and Singapore in 1986 [20]. Warmer temperature is associated with dengue incidence in Southeast Asia [21]. Most dengue transmission occurs when the temperature is higher than 20 °C. However, extremely high temperatures (>36 °C) can also limit the feeding activity of female mosquitoes to restrain transmission [16]. A series of statistical methods including negative binomial regression model, generalised additive model, times series were used in such studies. And due to the autocorrelation of dengue occurrence, times series model showed easy operation and powerful prediction ability in the actual application.

In this study, we use prewhitened cross-correlation and autoregressive integrated moving average (ARIMA) model to analyse the dengue occurrence in Guangzhou from 2001 to 2016. We seek to improve the understanding of critical risk factors for dengue transmission in Guangzhou, with the aim of developing a predictive dengue model to inform dengue prevention plans.

## Materials and methods

### Ethics statement

The present study was fully reviewed and approved by the Ethics Committee of the Guangzhou Center for Disease Control and Prevention (GZCDC) and the Ethics Committee of the School of Public Health of Sun Yat-sen University. All of the patient data were de-identified and the data were analysed anonymously.

### Research area

**Fig. 1.**
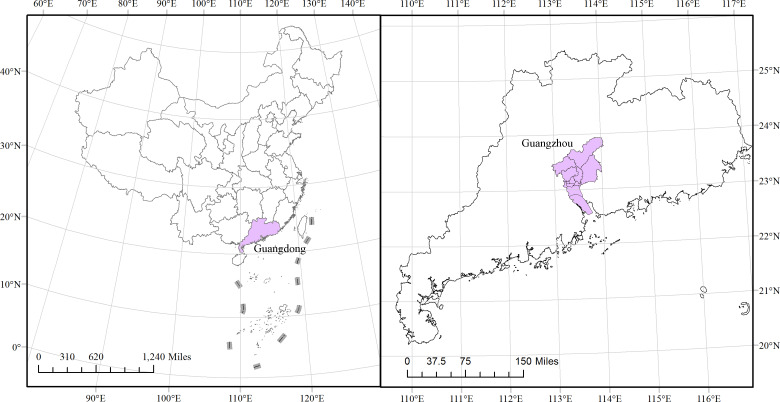
The location of Guangzhou in China. The left plot is the whole view of China, with a dark colour indicating Guangdong Province. The right graph zooms in on Guangdong Province, with a dark colour indicating its capital city Guangzhou.

Guangzhou is the capital city of Guangdong Province in southern China. With a current population of more than 12.84 million, it is one of the most highly populated cities in the world. Located at 112°57 E to 114°3 E and 22°26 N to 23°56 N and cover a total area of 7434.40 km^2^, Guangzhou has 10 administrative districts (Liwan, Yuexiu, Haizhu, Baiyun, Tianhe, Huangpu, Luogang, Panyu, Nansha and Huadu) and two satellite cities (Conghua and Zengcheng) (Fig. 1). Guangzhou is one of the most urbanised areas in China and is the most important center of international commerce in south China. It has a humid subtropical climate that is influenced by Asian monsoons [22], resulting in a wet and warm climate that is favourable for vector reproduction [10]. Over the past three decades, *Aedes albopictus* was observed to be the sole vector for dengue transmission in Guangzhou with the absence of *Aedes aegypti*. According to vector surveillance data, *Aedes albopictus* ranked second at a proportion of approximately 5.89% of all adult mosquitos, following only *Culex fatigans* (89.90%) in Guangdong province [23].

### Data sources

Monthly dengue cases aggregated by onset date from 2001 to 2016 were collected by the GZCDC in accordance with the Law on Prevention and Treatment of Infectious Diseases of China. Once diagnosed in medical institutions, the case must be reported to the web-based National Notifiable Infectious Disease Reporting Information System within 24 h according to the National Diagnostic Criteria for Dengue Fever (WS216-2008) published by the Chinese Ministry of Health [9]. Through the face-to-face interviews conducted by GZCDC, the cases are classified as either imported or indigenous based on the patient's travel history and dengue's human incubation period.

Monthly vector surveillance which was conducted in each midmonth days in total 36 representative communities, with the Breteau index (BI) for indoor larval density, standard space index (SSI) for outdoor larval density and adult mosquito density index (ADI) monitored by the GZCDC [24]. The definitions of the indices are as follows: BI = the number of containers positive for larvae or pupae per 100 houses, SSI = the number of containers positive for larvae or pupae per 100 standard spaces in which one stand space of 15 m^2^ outdoors and the ADI = the number of *Aedes* per hour by the human snare method. Monthly temperature and precipitation data for Guangzhou from 2001 to 2016 were downloaded from the China Meteorological Data Sharing Service System (http://cdc.nmic.cn/). The mean (*T*_mean_), maximum (*T*_max_) and minimum temperatures (*T*_min_) and the total (*P*_total_), mean (*P*_mean_) and maximum precipitation (*P*_max_) were used in this study.

### Data processing and time series analysis

The time series data collected above were divided into two parts as follows: the first 168 months (2001–2014) were used to construct the time series model with the Box and Jenkins approach [25, 26] and the last 24 months (2015–2016) was used to validate the predictive power of the model.

### ARIMA model for dengue occurrence

Because of temporal autocorrelations between data points and dengue's seasonal transmission patterns, time series analyses, especially ARIMA model, are effective to model dengue transmission. The model provides a general framework for analysing seasonal disease incidence data such as dengue [15], influenza [27], leptospirosis [28], tuberculosis [29], malaria [30] and brucellosis [31]. Accounting for temporal autocorrelations between variables can lead to higher accuracy using ARIMA on the same data [28]. Furthermore, it can incorporate external factors, such as climatic variables, which can improve the fitting and prediction accuracy; under these circumstances, the model as an extension of the ARIMA model is named the ARIMAX model.

The model expression ARIMA (*p*, *d*, *q*) (*P*, *D*, *Q*)_*s*_ [32], *p* is the order of the autoregression (AR) process, *q* is the number of moving average (MA) terms, *d* represents the differencing process to form a stationary times series, and *P*, *D* and *Q* are the seasonal orders of the AR, differencing and MA processes, respectively. Additionally, *s* denotes the seasonal period. Differencing was used to stabilise the variance and mean to fit the model's requirements [33]. In the ARIMA model, if the time series was nonstationary, the log transformation and differencing were used to remove the nonstationary terms. In our study, we generated a log transformation plus 2 (log (the number of indigenous cases +2)) for the monthly indigenous dengue cases. We added 2 to case incidence data to avoid zero prior to taking the log transformation and modeling processes. The predicted number of indigenous dengue cases at time *t* (*Y*_*t*_) was determined by the model equation (1) [26, 33]:1
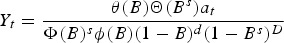
here, *θ*(*B*) is the operator for MA, Θ (*B*^*s*^) is the seasonal MA operator, *ϕ*(*B*) is the AR operator, Φ(*B*^*s*^) is the seasonal AR operator, (1 − *B*)^*d*^ and (1 − *B*^*s*^)^*D*^ denote the ordinary and seasonal difference components, respectively, *a*_*t*_ and *Y*_*t*_ represent the white noise and the dependent variable, respectively [33]. The *p* and *q* orders can be inferred using the cutoff time lag of the autocorrelation function (ACF) and the partial autocorrelation function (PACF). If the time series showed a seasonal pattern with an ACF peak at a time lag of *s*, then the same procedure was applied to the seasonal ARIMA model. The Ljung-Box test was used to test the model residuals did not show residual autocorrelation.

### Cross-correlation analysis

Due to strong autocorrelations in the data, correlations of the time series of the monthly number of indigenous cases with imported cases, vector densities and climate were difficult to identify. In this study, a prewhitening process was applied to the data among the multiple external regressors. Prewhitening to avoid common trends between incidence and risk factors [26], was conducted in three steps. First, we determined a time series model for the *y*-variable in step 1 and stored the residuals from this model and then filtered the *x*-variable series using the *y*-variable model in step 2 and finally examined the cross-correlation function (CCF) between the residuals from Step 1 and the filtered *x*-values from Step 2.

The CCF of the prewhitened imported cases, vector density and climate variables with the indigenous cases time series were calculated to identify the significant time lags. The factors that did not show a significant time lag not included in the model analysis [34]. Based on the results from previous studies as well as biological and epidemiological plausibility, we selected those positively significant lag values for the next step of the analysis with maximum lag steps of four.

### Univariate and multivariate ARIMAX analyses

The lag value of the statistically significant factors identified by the cross-correlation analysis was incorporated as external covariates into the ARIMAX model constructed above. The ARIMAX model is described by equation (2) [26, 33]:2
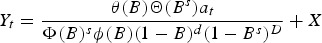
here, *X* is the external regressor, which may be univariate or multivariate. The other parameters are as described in the ARIMA model above. In this study, we first took a single lag value into the univariate ARIMAX model. For the multivariate analysis, the incorporated factors were selected from the factors with significant regression coefficients in the univariate analysis (*P* < 0.10). *P* < 0.05 in the regression coefficient test was considered statistically significant in the multivariate model. The coefficients of the model were estimated using the maximum likelihood method. The AIC was used to identify the best fitting model while taking into account possible overfitting of the data. In this study, RMSE was also used to determine the best performance. For the model diagnostics, the ARIMA and ARIMAX models were used to test whether model residuals were significantly different from white noise. Finally, we used cross-validation of one-step method using the selected model to measure model performance.

### Model validation for testing outbreak

According to the Technical Guidelines for Dengue Control and Prevention enacted by Chinese Center for Diseases Control and Prevention, a dengue outbreak is as the appearance of three confirmed indigenous cases in a period no longer than 15 days in a confined geographic area, such as one community, village, school or other collective units.

To investigate model robustness, the cross-validation for the testing outbreak was applied. However when applied to time series data traditional leave-one-out or k-fold cross validation may produce overly optimistic results due to temporal autocorrelation in the data, violating usual assumptions of independence. In our time series analysis, cross-validated error taking into account temporal dependence in the data was obtained as follows [35]: (1) Fit the model to the observed data *Y*_1_, *Y*_2_,…,*Y*_*t*_ and let 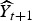 denotes the predicted value of the next observation. Then compute the error (*e*_*t*+1_ = *Y*_*t*+1_ − 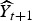) for the forecast observation. (2) Repeat step 1 for *t* = *k*,…, *n* − 1 where *k* is the minimum number of observations needed for fitting the model and *n* is the total number of observations. (3) Compute the mean RMSE from *e*_*k*+1_,…, *e*_*n*_. In our paper, we took *k* as 168 for our initial training data from January 2001 to December 2014 and the following 24 observations as testing data with initial forecast observation on January 2015 and the final forecast observation on December 2016.

The receiver operating characteristic curve (ROC) was used to determine the optimal cut-off point for the occurrence of an outbreak. Model accuracy was evaluated by the area under the ROC curve (AUC), larger values indicating higher accuracy. Afterwards the sensitivity, specificity and consistency rate for predicting outbreaks were computed, based on evaluating model error on testing data from January 2015 to December 2016 [36].

The R software (version 3.2.2, the R foundation for Statistical Computing, Vienna, Austria) with the TSA, forecast and pROC packages were used for the time series analysis and graphical display.

## Results

### Dengue occurrence patterns and their associated factors

**Fig. 2.**
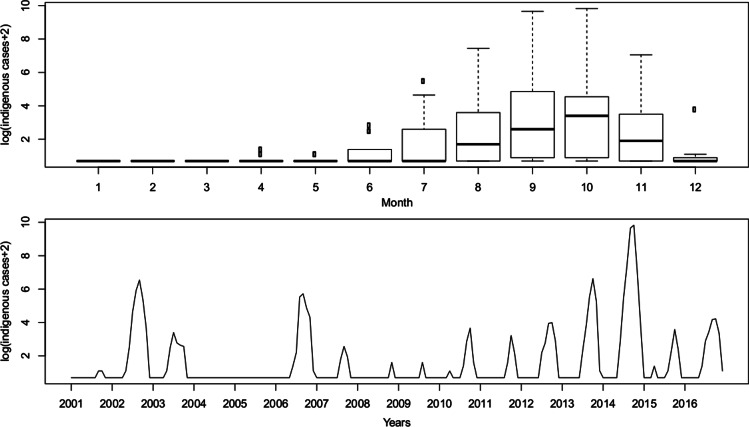
Natural logarithm of the monthly indigenous dengue cases +2 from 2001 to 2016 in Guangzhou, China.

A total of 42 470 dengue cases were reported from 2001 to 2016, including 41 370 (97.41%) indigenous cases and 1100 (2.59%) imported cases. The dengue transmission season was generally restricted to the period between summer (June–August) and autumn (September–November), with a peak in September. There were 4 years of major local outbreaks consisting of 1422 cases in 2002, 765 cases in 2006, 1249 cases in 2013 and 37 340 cases in 2014. To stabilise the variance and subsequent differencing of indigenous dengue case incidence data, we calculated the natural log transformation plus 2 (log (number of indigenous cases +2)) for the monthly indigenous cases in the study, which is depicted in Figure 2.

**Fig. 3.**
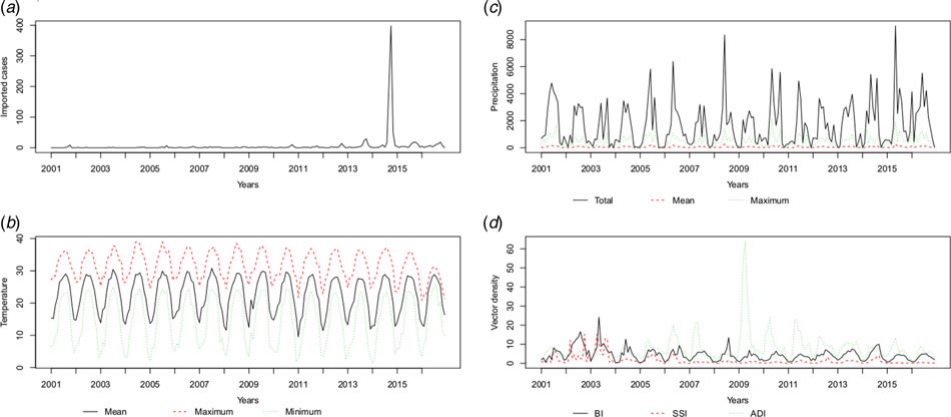
The patterns of the recorded risk factors, including (a) imported cases (n), (b) temperature (°C), (c) precipitation (10* mm) and (d) vector density (percentages for BI and SSI, numbers per hour for ADI) per month.

The temperature (*T*_mean_, *T*_max_ and *T*_min_), precipitation (*P*_total_, *P*_max_ and *P*_mean_) and vector density (BI, SSI and ADI) variables showed similar patterns, with high values in the summer and autumn. The average monthly number of imported cases over the study period is plotted in Figure 3 and Supplementary Table S1. A monthly number of imported cases generally proceeded indigenous cases, which arose through the local transmission. The highest total precipitation (*P*_total_ = 3006.5 mm) occurred in 2016, although there were 204 reported indigenous cases in that year; meanwhile, the lowest total precipitation (*P*_total_ = 1338.7 mm) occurred in 2003, when there were 76 reported indigenous cases.

### ARIMA model

**Fig. 4.**
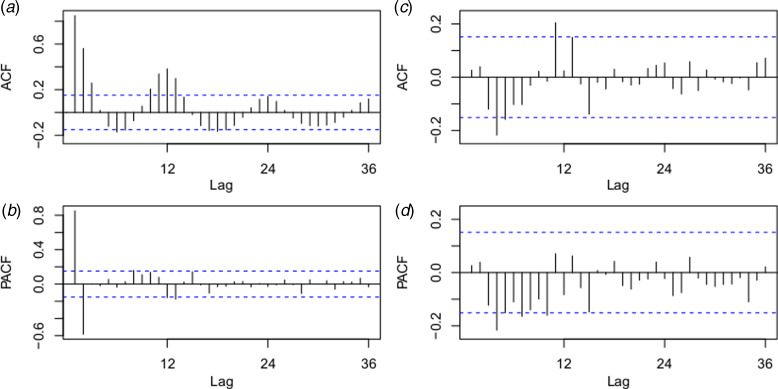
Autocorrelation function (ACF) and partial ACF (PACF) plots of the log transformation plus 2 for the original monthly indigenous dengue cases (a and b) and the ACF and PACF plots for the residuals of the ARIMA (0,1,1)(0,0,2)_12_ model (c and d). The dotted lines indicate the 95% confidence intervals.

The best fitting model selected according to AIC was the ARIMA(0,1,1)(0,0,2)_12_ model with an AIC of 400.8343 and RMSE of 0.7762. The ACF and PACF plots were used to estimate the temporal autocorrelation, as demonstrated in Figure 4. The Ljung-Box test indicated that model residuals did not significantly depart from a white noise process (*P* > 0.05).

### Cross-correlation analysis

After prewhitening, we calculated the cross-correlation coefficients between the mosquito densities, climate variables and imported cases. Table 1 shows that the log(indigenous cases + 2) time series correlated positively with the imported cases at the 0 and 1-month lags, the mean temperature at lag 0, the maximum temperature at lag 0 and the minimum temperature at lag 0.

**Table 1.** Cross-correlations between the prewhitened factors and the indigenous cases (log (number of indigenous cases +2)) time series between 2001 and 2014

	lag0	lag1	lag2	lag3	lag4
ICs	0.3191	0.1719	0.0202	0.0155	−0.1222
	+	+	.	.	.
BI	0.1253	0.0520	−0.1098	−0.1934	−0.2046
	.	.	.	−	−
SSI	−0.0384	0.0745	0.0295	−0.0590	−0.1953
	.	.	.	.	−
ADI	0.0540	0.0204	−0.0443	−0.1707	−0.1672
	.	.	.	−	−
*T*_mean_	0.2915	0.1077	−0.2140	−0.3616	−0.4282
	+	.	−	−	−
*T*_max_	0.2608	0.1410	−0.2327	−0.2918	−0.3789
	+	.	−	−	−
*T*_min_	0.2641	0.0419	−0.2117	−0.3239	−0.3487
	+	.	−	−	−
*P*_total_	−0.0418	−0.0779	−0.0872	−0.1434	−0.0865
	.	.	.	.	.
*P*_mean_	−0.0390	−0.0781	−0.0864	−0.1411	−0.0881
	.	.	.	.	.
*P*_max_	−0.0126	−0.0443	−0.0766	−0.1569	−0.0216
	.	.	.	−	.

ICs, imported cases; BI, Breteau index; SSI, standard space index; ADI, adult density index; *T*_mean_, mean temperature; *T*_max_, maximum temperature; *T*_min_, minimum Temperature; *P*_total_, total precipitation; *P*_mean_, mean precipitation; *P*_max_, maximum precipitation.

‘+’, positive statistical significance; ‘.’ no statistical significance; ‘−’, negative statistical significance.

### Univariate and multivariate ARIMAX analyses

Based on the previous cross-correlation analysis, we tested the ARIMAX model by incorporating the imported cases at lag 0 and lag 1, mean temperature at lag 0, maximum temperature at lag 0 and minimum temperature at lag 0 as external regressors based on the ARIMA (0,1,1)(0,0,2)_12_ model from a log (indigenous cases +2) time series.

In the fitting process, the imported cases at lag 0 and the minimum temperature at lag 0 were statistically significant in the univariate analysis. As shown in Table 2, the ARIMAX (0,1,1)(0,0,2)_12_ model with the imported cases had the lowest AIC (395.9477) and fitting RMSE (0.7607) in the fitted model and the lower validation RMSE (0.6813) compared with the ARIMAX (0,1,1)(0,0,2)_12_ model with the minimum temperature. In the multivariate analysis, both the imported cases at lag 0 and minimum temperature at lag 0 were statistically significant, as demonstrated in Table 3. The ACF and PACF for the residuals of the ARIMAX (0,1,1)(0,0,2)_12_ model with the imported cases and the ARIMAX (0,1,1)(0,0,2)_12_ model incorporating both the imported cases and minimum temperature at lag 0 were depicted in Supplementary Figure S1.

**Table 2.** Summary of the fitted parameters of the univariate ARIMAX model analysis in Guangzhou, 2001–2016

Model	Fitted		Test RMSE	Risk factors				
	AIC	RMSE		Vars	Coef.	s.e.	*T*	*P*-value
ARIMA(0,1,1)(0,0,2)_12_	400.8343	0.7762	0.6820					
				ma1	0.3871	0.0712	5.4363	<0.0001**
				sma1	0.3863	0.0897	4.3075	<0.0001**
				sma2	0.2110	0.0710	2.9727	0.0034**
ARIMAX(0,1,1)(0,0,2)_12_ with ICs	395.9477	0.7607	0.6813					
				ma1	0.3633	0.0720	5.0461	<0.0001**
				sma1	0.3645	0.0853	4.2746	<0.0001**
				sma2	0.2115	0.0700	3.0206	0.0029**
				ICs(lag0)	0.0045	0.0017	2.6313	0.0093**
ARIMAX(0,1,1)(0,0,2)_12_ with ICs	399.2842	0.7742	0.6966					
				ma1	0.3872	0.0714	5.4200	<0.0001**
				sma1	0.3814	0.0924	4.1260	<0.0001**
				sma2	0.2084	0.0720	2.8930	0.0043*
				ICs(lag1)	0.0004	0.0018	0.2160	0.8293
ARIMAX(0,1,1)(0,0,2)_12_ with *T*_mean_	400.7511	0.7720	0.6847					
				ma1	0.3686	0.0724	5.0915	<0.0001**
				sma1	0.3679	0.0909	4.0479	0.0001**
				sma2	0.1946	0.0714	2.7234	0.0071**
				*T*_mean_(lag0)	0.0336	0.0234	1.4376	0.1524
ARIMAX(0,1,1)(0,0,2)_12_ with *T*_max_	400.6336	0.7712	0.6974					
				ma1	0.3681	0.0725	5.0769	<0.0001**
				sma1	0.3786	0.0913	4.1485	0.0001**
				sma2	0.2137	0.0715	2.9897	0.0032**
				*T*_max_(lag0)	0.0372	0.0253	1.4670	0.1443
ARIMAX(0,1,1)(0,0,2)_12_ with *T*_min_	399.3669	0.7686	0.6817					
				ma1	0.3713	0.0711	5.2197	<0.0001**
				sma1	0.3915	0.0888	4.4086	<0.0001**
				sma2	0.1791	0.0748	2.3951	0.0177*
				*T*_min_(lag0)	0.0322	0.0173	1.8656	0.0638*

ARIMAX, autoregressive integrated moving average with external regressors; Fitted, fitted results; Test, test results; AIC, Akaike Information Criterion; RMSE, root mean square error; Coef., coefficient of risk factors; lag, time lag of risk factors; s.e., standard error; *t*, *t* statistic; ma1, MA(1); sma1, seasonal MA(1); sma2, seasonal MA(2).

**P < 0.01, *P < 0.10.

**Table 3.** Summary of the fitted parameters of the multivariate ARIMAX model analysis in Guangzhou, 2001–2016

Model	Fitted		Test RMSE	Risk factors				
	AIC	RMSE		Vars	Coef.	s.e.	*T*	*P-*value
ARIMAX(0,1,1)(0,0,2)_12_ with ICs and *T*_min_	393.7854	0.7520	0.6445					
				ma1	0.3472	0.0714	4.8636	<0.0001**
				sma1	0.3631	0.0842	4.3105	<0.0001**
				sma2	0.1730	0.0745	2.3207	0.0215*
				ICs (lag0)	0.0047	0.0017	2.7640	0.0064**
				*T*_min_ (lag0)	0.0347	0.0170	2.0484	0.0421*

ARIMAX, autoregressive integrated moving average with external regressors; Fitted, fitted results; Test, test results; AIC, Akaike Information Criterion; RMSE, root mean square error; Coef., coefficient of risk factors; lag, time lag of risk factors; s.e., standard error; *t*, *t* statistic; ma1, MA(1); sma1, seasonal MA(1); sma2, seasonal MA(2).

**P < 0.01, *P < 0.05.

**Fig. 5.**
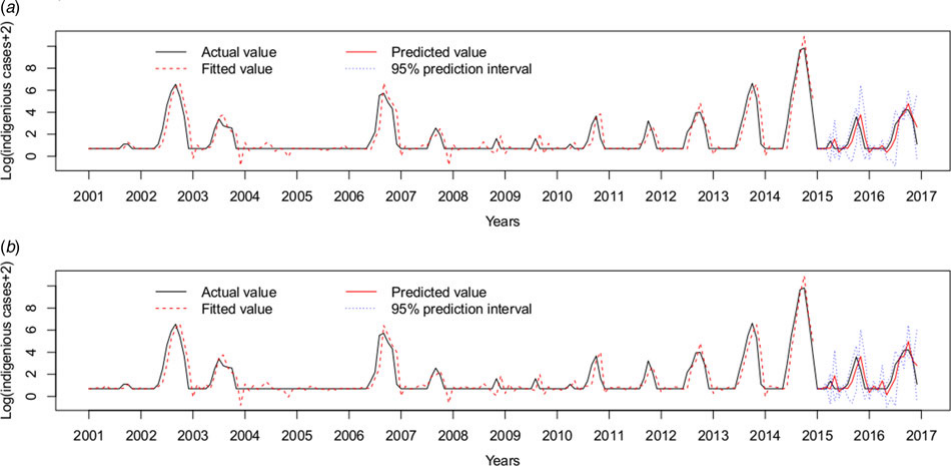
Log transformation of the monthly dengue indigenous cases in Guangzhou from 2001 to 2016. (a) ARIMAX (0,1,1)(0,0,2)_12_ model with the imported cases at lag 0 tested by one-step validation method for the test data, (b) ARIMAX (0,1,1)(0,0,2)_12_ model with both the imported cases and the minimum temperature at lag 0, predicted by one-step validation method by test data.

Therefore, the ARIMAX (0,1,1)(0,0,2)_12_ model incorporating both the imported cases and the minimum temperature at lag 0 was the best fitting model, with the lowest AIC (393.7854) and RMSE of 0.7520. The validation RMSE (0.6445) was lower than for the ARIMAX (0,1,1)(0,0,2)_12_ model with the imported cases at lag 0(0.6813), an decrease of 5.40% (Fig. 5).

### Model validation for testing outbreak

**Fig. 6.**
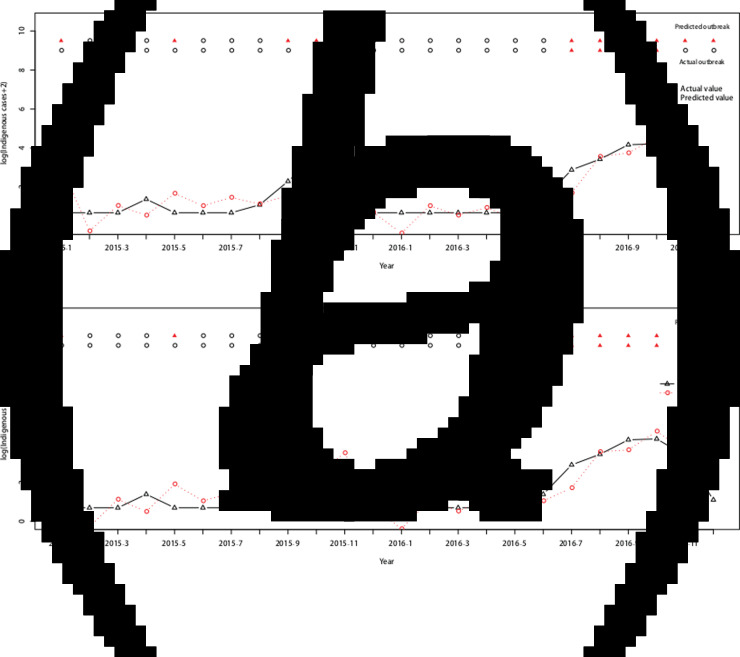
Cross-validation for testing dengue outbreak in Guangzhou. The upper row of points in the graph denotes the predicted outbreaks by our model and the lower row shows the observed outbreaks. The lines show the predicted values and actual values respectively. (a) Testing performance for the ARIMAX (0,1,1)(0,0,2)_12_ model with the imported cases at lag 0. (b) Testing performance for the ARIMAX (0,1,1)(0,0,2)_12_ model with both the imported cases and minimum temperature at lag 0.

Forty-five (23.44%) outbreaks were identified among the 192 observations from January 2001 to December 2016. On the training dataset from January 2001 to December 2014, the AUC for the ARIMAX (0,1,1)(0,0,2)_12_ model including imported cases at lag 0 was 0.9719 with the best threshold as 1.5524 and the AUC for the ARIMAX (0,1,1)(0,0,2)_12_ model including both imported cases and minimum temperature at lag 0 was 0.9754 with the best threshold as 1.6096 (Supplementary Fig. S2). On the testing dataset (testing outbreak), the ARIMAX(0,1,1)(0,0,2)_12_ model with imported cases at lag 0 had a sensitivity of 1.0000 (1/1), a specificity of 0.6842(13/19) and a consistency rate of 0.7500(18/24). The ARIMAX(0,1,1)(0,0,2)_12_ incorporating both the imported cases and minimum temperature at lag 0 had a sensitivity of 1.0000 (1/1), a specificity of 0.7368 (14/19) and a consistency rate of 0.7917 (19/24). More details can be found in Figure 6 and Supplementary Table S2.

## Discussion

In this study, we developed and evaluated time series models to characterise the risk factors of local dengue transmission in Guangzhou to inform control measures. We found that the number of imported cases and the minimum temperature were the important variables associated with local dengue transmission in Guangzhou City. And the multivariate ARIMAX model that included both the imported cases and the minimum temperature at lag 0 had a better fit than the inclusion of one variable alone. This modeling technique can be a useful tool for planning local control interventions and could be implemented during routine dengue surveillance in Guangzhou.

A higher number of overseas travellers boost the risk of global dengue transmission [37]. Previous studies of phylogenetic analysis and travel history surveys suggest that imported cases could affect dengue transmission [9, 38]. However, few studies have quantitatively modeled imported cases with dengue occurrence. In the unprecedented 2014 dengue outbreak in Guangzhou, the number and timing of the imported dengue cases strongly determined the local occurrence and outbreak size [10]. Virus dissemination due to population movement contributes to an increase in the outbreak potential in non-endemic areas [33]. Increased tourism and commerce can introduce the dengue virus into non-endemic areas or new districts. Currently, dengue is endemic in Southeast Asian countries such as Thailand, Malaysia, Philippines, Cambodia, Laos, Vietnam, Indonesia and India. The high frequency of international travel and large shipping industry in Guangzhou increases the risk of importation of dengue cases which can lead to indigenous epidemics.

Temperature also influences the dengue transmission dynamics, mainly through indirect effects. Increasing temperatures can increase the biting frequency of *Aedes* and the survival rates during emergence [39] and reduce the extrinsic incubation period of dengue in the mosquito [40]. However, there is little evidence that the highest temperature drives patterns of dengue epidemics in Southeast Asia [21]. Interestingly, the minimum temperature played a significant role in dengue transmission in this study. Several studies have highlighted the association between the minimum temperature and dengue transmission. In Taiwan, the minimum temperature correlated with dengue cases at a lag of 1–3 months [41]. In Guadeloupe, minimum temperature with a 5 week time lag was the best climatic variable for the prediction of dengue outbreaks [33]. Thus, the evidence suggests that more severe dengue transmission occurs at higher monthly minimum temperature.

Prewhitening was conducted prior to calculating the cross-correlation between the dengue cases and risk factors. This process can measure the association between temporally auto-correlated data, whereas the Pearson or Spearman correlation requires independent and identically distributed observations [15]. Finally, we found that dengue cases significantly correlated with both the number of imported cases and the minimum temperature at lag 0. However, no positive correlation was found with the BI, SSI, ADI and precipitation at any lag. The mean, maximum and minimum temperatures all showed a strong correlation with the number of dengue cases at lag 0. The models incorporating these variables as external regressors indicated improved model performance [41]. At present, it is generally known that the increasing risk of dengue transmission is dependent on vector density, however, its correlation with dengue is generally poor, as shown in our study [15]. Excepting for vector density, this phenomenon may in part be due to the fact, vector capacity is also related with biting rate, the probability of vector survival, extrinsic incubation period and vector competence [42]. This result may also be associated with the method itself due to the subjectivity of the vector density survey and potential bias in data collection. Especially for adult mosquito density, which theoretically shows a higher correlation with dengue risk than larval indices because only the adult life stage directly contributes to dengue transmission dynamics was also in poor association with dengue incidence.

Our study provides deep insights into the dengue risk in Guangzhou through the characterization of the temporal autocorrelation structure. The results of this study are consistent with previous research showing that the number of imported cases is highly correlated with local transmission in Guangzhou [9, 10]. The methodology used in the study is an effective complement for understanding dengue risk factors in the region of Guangzhou and can be applied in high-risk areas of south China. As the imported cases are highly associated with local transmission, port inspection and quarantine may be able to play a critical role in dengue control in the region. The quarantine mainly targets fever cases (⩾38 °C) entering Guangzhou by plane, with the serum collected and polymerase chain reaction laboratory test followed. Approximately 30% of the total imported cases were identified by port quarantine in Guangzhou. When imported cases escaped detection by port monitoring systems, the rapid rate of case detection in local health institutions is also important in control practices, which can be improved by training to increase knowledge, attitudes and practices of both health workers and the public [43]. When the minimum temperature arises, the vector control efforts should be strengthened, especially in the area with a high likelihood of imported cases. Due to Guangzhou as the imported dengue area, the strategy under this study is an economic and effective way to prevent dengue local outbreak.

Admittedly, there were limitations in our study. The ARIMAX model can only identify a correlation between local dengue incidence and the risk variables but cannot identify causal relationships [27]. Furthermore, our study did not take into account the effects of human movement [14], water supply [13], land utilisation [44] and socio-economic factors [45]. These factors also play certain roles in determining dengue transmission in the study regions. Due to the experiences and lessons from 2014, Guangzhou authorities took strict intervention measures, especially in hospital isolation and early diagnosis using the NS1 antigen test [46], to successfully reduce the number of local cases during the 2015 transmission season. Finally, due to the lag 0 for both factors including in the models, it is reasonable for fitting the model conforming to the actual public health practices. Using time interval with monthly data, when an imported case is introduced, it will cost longest 20 days roundly to disperse, including an incubation period about 3–15 day and longest infectious period 5 days after onset which is less than 1 month. Therefore, the introduced imported case in a current 1 month is more important. Furthermore, it is a bit delayed to use the onset date for monthly aggregation analysis instead of the infection date or biting date. However, it would be more objective if the accurate date could be confirmed.

The frequency and scale of dengue transmission in China have increased in recent years [8]. A better understanding of the relationship between dengue occurrence and various risk factors can be used to prevent further large outbreaks. Our ARIMAX modelling provides a practical approach for monitoring dengue in Guangzhou City. The model and its outcome can give objective guidance for local health authorities in order to better understand future large epidemics.

## Supplementary material

For supplementary material accompanying this paper visit https://doi.org/10.1017/S0950268818001176. click here to view supplementary material
