# Supplementary material for: Imported cases and minimum temperature drive dengue transmission in Guangzhou, China: evidence from ARIMAX model
Source: Epidemiol Infect. 2018 May 21;146(10):1226–35. doi: 10.1017/S0950268818001176 (PMC9134281; doi:10.1017/S0950268818001176)
Supplement: Supplementary file 1 [file S0950268818001176sup001.zip › S0950268818001176sup001/Supplementary_Table_S1.docx]

**Supplementary Table S1.** Monthly descriptive statistics of the dengue cases and risk factors, 2001-2016

| Variables (unit) | Range | Mean$\pm$SD |
| --- | --- | --- |
| Indigenous cases (n) | 0.00-18557.00 | 215.47 $\pm$ 1750.78 |
| ICs (n) | 0.00-398.00 | 5.73 $\pm$ 32.49 |
| BI | 0.23-24.17 | 4.41 $\pm$ 3.22 |
| SSI | 0.00-15.26 | 1.48 $\pm$ 2.50 |
| ADI | 0.60-63.86 | 7.91 $\pm$ 7.40 |
| T_mean_ (°C) | 9.52-30.77 | 22.45 $\pm$ 5.60 |
| T_max_ (°C) | 20.81-39.10 | 31.65 $\pm$ 4.33 |
| T_min_ (°C) | 1.30-25.79 | 14.54 $\pm$ 7.48 |
| P_total_ (mm) | 0.00-901.60 | 169.66 $\pm$ 163.72 |
| P_mean_ (mm) | 0.00-29.08 | 5.56 $\pm$ 5.35 |
| P_max_ (mm) | 0.00-214.70 | 47.49 $\pm$ 38.21 |

ICs: imported cases, BI: Breteau Index, SSI: Standard Space Index, ADI: Adult Density Index, T_mean_: mean temperature, T_max_: maximum temperature, T_min_: minimum temperature, P_total_: total precipitation, P_mean_: mean precipitation, P_max_: maximum precipitation. n: the number of cases.
