# Supplementary material for: Imported cases and minimum temperature drive dengue transmission in Guangzhou, China: evidence from ARIMAX model
Source: Epidemiol Infect. 2018 May 21;146(10):1226–35. doi: 10.1017/S0950268818001176 (PMC9134281; doi:10.1017/S0950268818001176)
Supplement: Supplementary file 1 [file S0950268818001176sup001.zip › S0950268818001176sup001/Supplementary_Table_S2.docx]

Supplementary Table S2. The outbreak predicting performance for the models

| Model | Threshold | Sensitivity | Specificity | Consitency rate |
| --- | --- | --- | --- | --- |
| 1* | 1.5524 | 1.0000 | 0.6842 | 0.7500 |
| 2* | 1.6096 | 1.0000 | 0.7368 | 0.7917 |

* Model1:the ARIMAX (0,1,1)(0,0,2)_12_ model with the imported cases at lag 0. Model 2: the ARIMAX (0,1,1)(0,0,2)_12_ model with both the imported cases and minimum temperature at lag 0.
