# Supplementary figures and images for: Imported cases and minimum temperature drive dengue transmission in Guangzhou, China: evidence from ARIMAX model
Source: Epidemiol Infect. 2018 May 21;146(10):1226–35. doi: 10.1017/S0950268818001176 (PMC9134281; doi:10.1017/S0950268818001176)

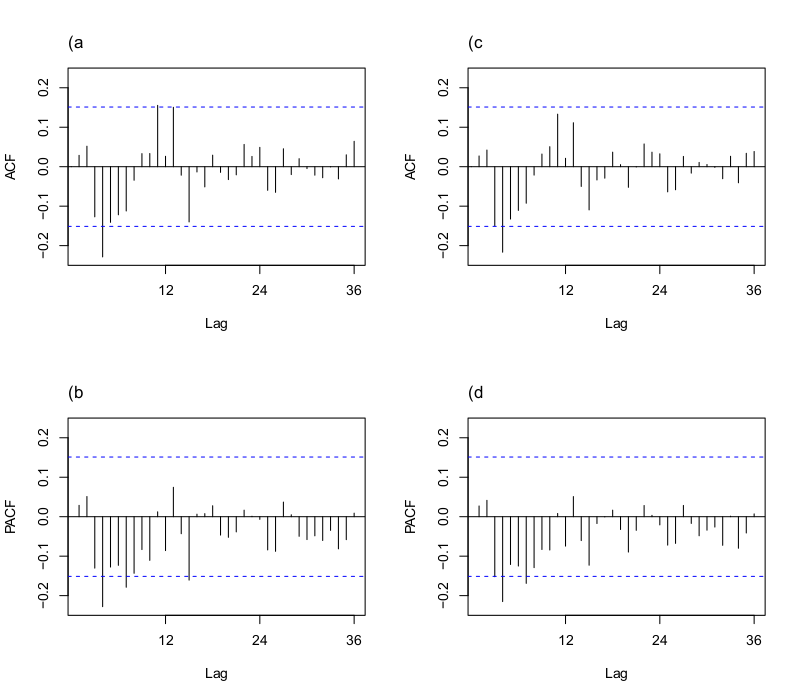

Supplement: Supplementary file 1 [file S0950268818001176sup001.zip › S0950268818001176sup001/Supplementary_Fig._S1.tif]

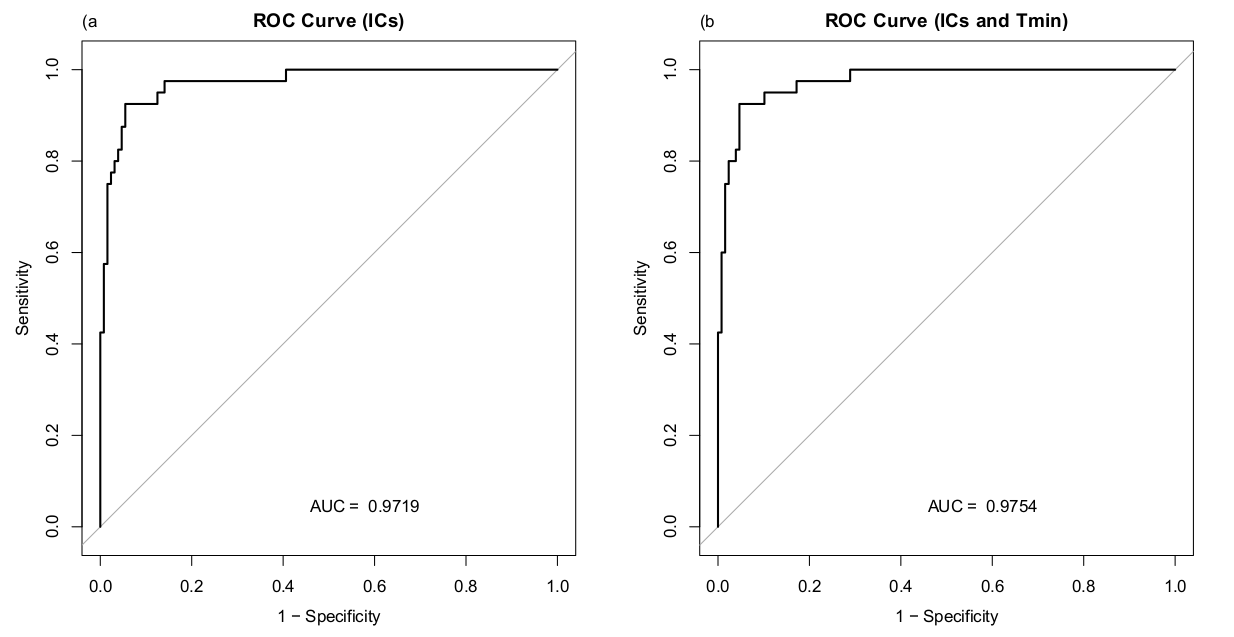

Supplement: Supplementary file 1 [file S0950268818001176sup001.zip › S0950268818001176sup001/Supplementary_Fig._S2.tif]
